# Melatonin Inhibits GnRH-1, GnRH-3 and GnRH Receptor Expression in the Brain of the European Sea Bass, *Dicentrarchus labrax*

**DOI:** 10.3390/ijms14047603

**Published:** 2013-04-08

**Authors:** Arianna Servili, Patricia Herrera-Pérez, María del Carmen Rendón, José Antonio Muñoz-Cueto

**Affiliations:** 1 Department of Biology, Faculty of Marine and Environmental Sciences, University of Cadiz, Marine International Campus of Excellence (CEI·MAR), University Campus of Puerto Real, Puerto Real E-11510, Spain; E-Mails: patricia.herrera@uca.es (P.H.-P.); maricarmen.rendon@uca.es (M.C.R.); 2 Andalusian Center of Marine Sciences and Technologies (CACYTMAR), Research Institutes, University Campus of Puerto Real, Puerto Real E-11510, Spain

**Keywords:** sea bass, melatonin, GnRH, GnRH receptors, brain, reproduction

## Abstract

Several evidences supported the existence of melatonin effects on reproductive system in fish. In order to investigate whether melatonin is involved in the modulation of GnRH systems in the European sea bass, we have injected melatonin (0.5 μg/g body mass) in male specimens. The brain mRNA transcript levels of the three GnRH forms and the five GnRH receptors present in this species were determined by real time quantitative PCR. Our findings revealed day–night variations in the brain expression of GnRH-1, GnRH-3 and several GnRH receptors (dlGnRHR-II-1c, -2a), which exhibited higher transcript levels at mid-light compared to mid-dark phase of the photocycle. Moreover, an inhibitory effect of melatonin on the nocturnal expression of GnRH-1, GnRH-3, and GnRH receptors subtypes 1c, 2a and 2b was also demonstrated. Interestingly, the inhibitory effect of melatonin affected the expression of hypophysiotrophic GnRH forms and GnRH receptors that exhibit day–night fluctuations, suggesting that exogenous melatonin reinforce physiological mechanisms already established. These interactions between melatoninergic and GnRH systems could be mediating photoperiod effects on reproductive and other rhythmic physiological events in the European sea bass.

## 1. Introduction

Melatonin (*N*-acetyl-5-methoxytryptamine) is an indolamine mainly synthesized by the pineal organ that is involved in the synchronization of many rhythmic physiological processes to the environmental cues [1–4]. In non-mammalian vertebrates melatonin works both as a clock, since the duration of the plasma melatonin rise corresponds to the length of the night, and as a calendar, because the seasonal changes in the length of the days and temperature modulates the duration and amplitude of the nocturnal melatonin increase, respectively [5–7].

To date, many evidences have shown a role of melatonin in fish reproduction, by acting at different levels of the reproductive axis. Thus, melatonin has stimulatory effects on brain dopaminergic system in eel [8]. Moreover, in the Atlantic croaker melatonin treatment increases luteinizing hormone levels, by activating the preoptic/hypothalamic areas and the pituitary gland [9]. Further evidences showing the pituitary gland as a target for melatonin actions are the presence of hypophysary melatonin receptors and/or actions in several fish species, including sea bass [10–13]. Melatonin binding sites and/or receptors were also present in the gonads of sea bream [5], carp *Catla catla*[14], sea bass [15] and some mammals [16–18]. However, available information led to conflicting conclusions regarding the role of melatonin in fish reproductive events. There are some findings showing that melatonin administration can induce stimulatory [9,19,20] or inhibitory [21,22] effects, depending on the melatonin dose, the age, the reproductive stage of animals and experimental design.

The European sea bass (*Dicentrarchus labrax*) is a seasonal species having a natural spawning period during winter that can be modified by modulating photoperiod and temperature regimes [20]. As in other vertebrates, the reproductive process in sea bass is governed by the stimulatory actions of gonadotrophin-releasing hormone (GnRH). In sea bass, these GnRH systems have been studied in detail in recent years, evidencing the expression of three different GnRH forms, *i.e.*, GnRH-1, GnRH-2 and GnRH-3 [23–27] that could act through five different GnRH receptors [28–31]. Several evidences suggest that melatonin could be interacting with the reproductive axis in sea bass. Daily and seasonal rhythms of melatonin secretion [7,32,33] and melatonin binding sites [34,35] have been revealed in sea bass. Moreover, melatonin receptors are expressed in all elements of the sea bass reproductive axis, *i.e.*, the neuroendocrine brain, the pituitary and the gonads [13,36]. Some reproductive hormones, including GnRH, exhibit daily [37] and/or seasonal variations [38–41]. In addition, changes in photoperiod conditions can influence reproductive performance and puberty in sea bass by altering circadian and seasonal variations of reproductive hormones, as well as melatonin content and/or melatonin binding sites [34,35,42].

The major aim of this work is to investigate the role of melatonin on the reproductive axis and, in particular, the relationship between the melatoninergic and GnRH systems, in the European sea bass. For this purpose, we have analysed the daily variations in the brain expression of different GnRH forms and GnRH receptors present in the European sea bass by real time quantitative PCR. Moreover, we have reported the effects of the exogenous administration of melatonin on all these elements of the sea bass GnRH system.

## 2. Results

**Figure 1:**
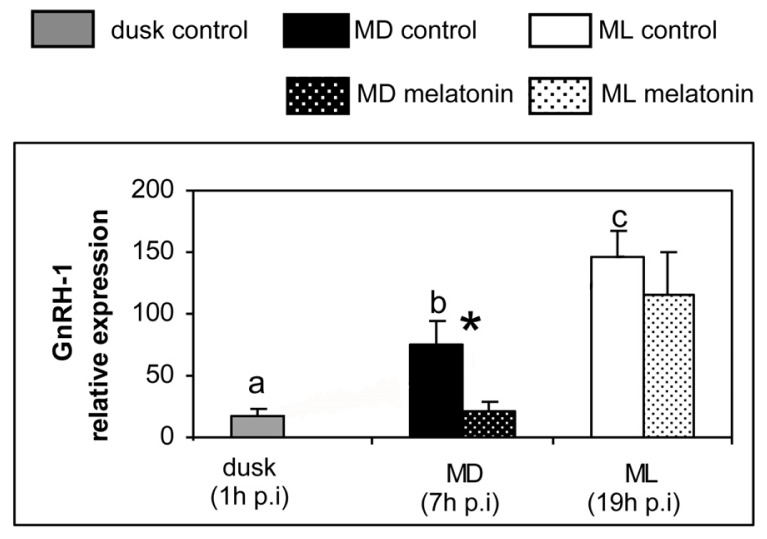

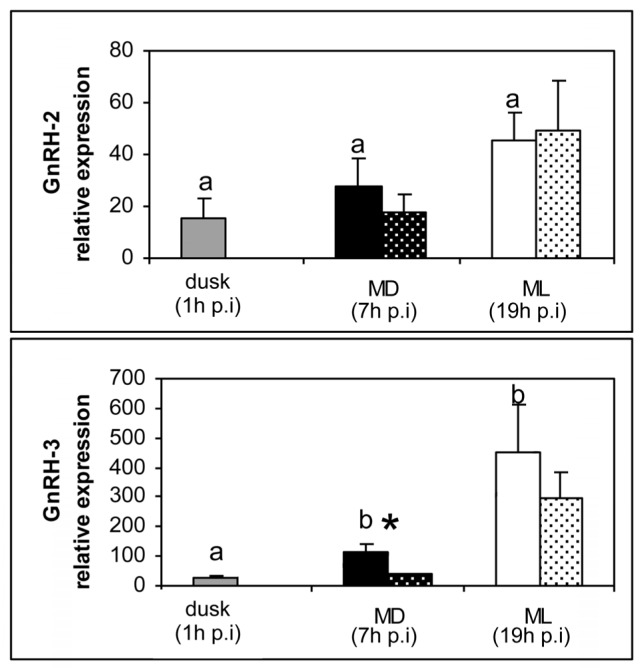
Relative expression of different *GnRH* forms determined by quantitative real-time PCR in sea bass injected with vehicle (plain columns) or melatonin (dotted columns). The expression of the control group at dusk (one hour after the injection) was used as the reference start point. Gene expression was also determined at mid-dark (MD, seven h after the injection) and at mid-light (ML, nineteen hours after the injection) phases of the photocycle in both control and melatonin groups (*n* = 7). Statistical daily variation among control animals was determined by one way ANOVA followed by LSD test. There are no statistical differences among groups that share common letters. Differences in the expression between the control and the melatonin-treated group at MD and ML were determined by two way ANOVA analysis followed by LSD test. Non-parametric Kruskal-Wallis ANOVA on ranks was used when homogeneity of variances and normality of data were not accomplished. Asterisks indicate significant difference between control and melatonin-treated groups. Significant differences were considered at *p* < 0.05. The expression of *18S* gene was used for normalization. Abbreviations: p.i., post-injection.

**Figure 2:**
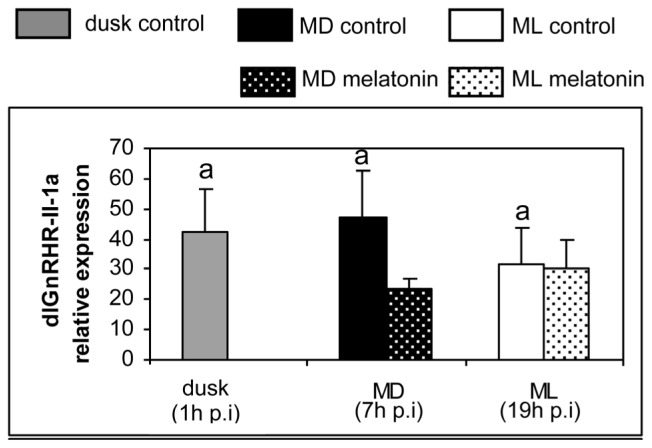

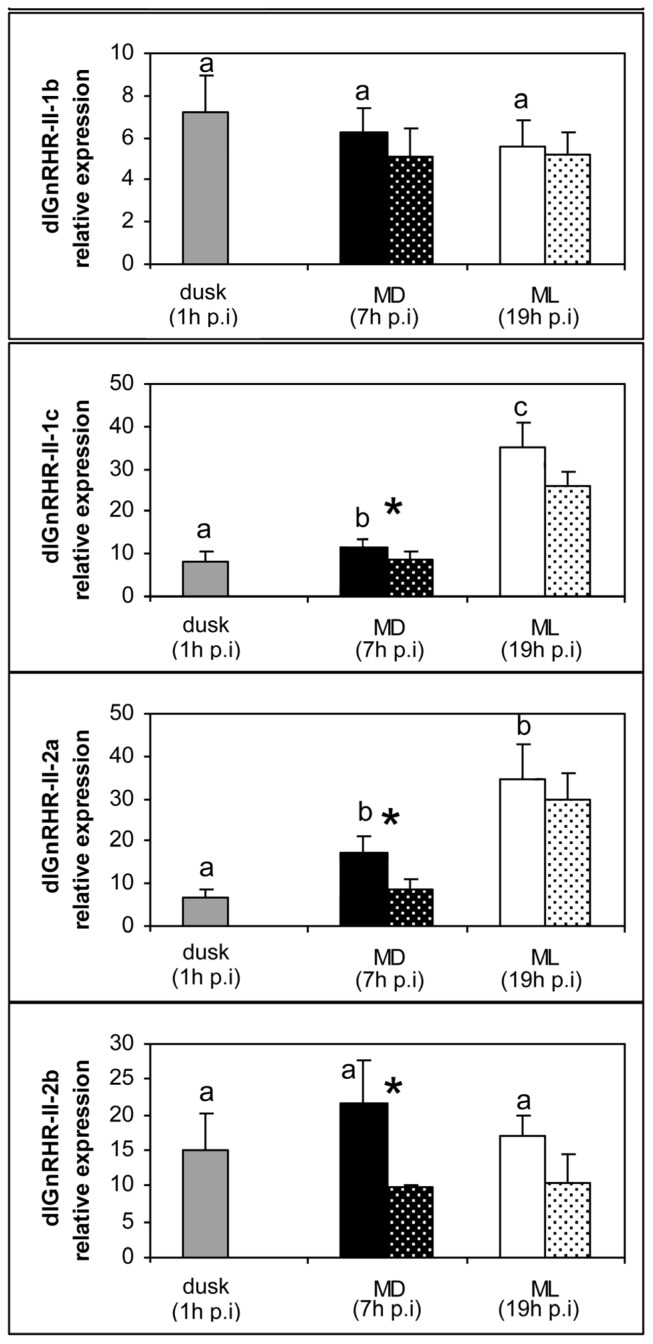
Relative expression of different *GnRH* receptor subtypes determined by quantitative real-time PCR in sea bass injected with vehicle (plain columns) or melatonin (dotted columns). The expression of the control group at dusk (one hour after the injection) was used as the reference start point. Gene expression was also determined at mid-dark (MD, seven h after the injection) and at mid-light (ML, nineteen hours after the injection) phases of the photocycle in both control and melatonin-treated groups. Statistical daily variation among control animals was determined by one way ANOVA followed by LSD test. There are no statistical differences among groups that share common letters. Differences in the expression between the control and the melatonin-treated group at MD and ML were determined by two way ANOVA analysis followed by LSD test. Non-parametric Kruskal-Wallis ANOVA on ranks was used when homogeneity of variances and normality of data were not accomplished. Asterisks indicate significant difference between control and melatonin-treated groups. Significant differences were considered at *p* < 0.05. The expression of *18S* gene was used for normalization. Abbreviations: p.i., post-injection.

The results obtained are illustrated in Figures 1 and 2. Our quantitative real-time PCR analysis in sea bass brain revealed the existence of day–night variations in the expression of *GnRH-1* and *GnRH-3* in the control group (Figure 1). In both cases mRNA expression was lower at dusk and increased significantly at mid-dark (MD), exhibiting the highest transcript levels at mid-light phase of the photocycle (ML, Figure 1). In contrast, no significant daily variation was observed for *GnRH-2* expression in control animals (Figure 1). Melatonin injection decreased significantly the expression of *GnRH-1* and *GnRH-3* at MD (7 h post-injection), but no significant differences were observed between controls and melatonin-injected animals at ML (19 h post-injection, Figure 1). No melatonin effect on *GnRH-2* expression was observed either at MD or at ML (Figure 1).

Regarding the brain expression levels of the *GnRH* receptors, we have observed a significant daily variation in the control group for the *dlGnRHR-II-1c* and *dlGnRHR-II-2a* receptor subtypes, with significantly higher values during the day (ML) in relation to night (MD and/or dusk, Figure 2). No apparent day–night variation was observed for the remaining receptor subtypes (*dlGnRHR-II-1a*, *-1b*, *-2b*). Melatonin injection reduced the expression of *dlGnRHR-II-1c*, *dlGnRHR-II-2a* and *dlGnRHR-II-2b* receptors significantly at MD (7 h post-injection, Figure 2). No melatonin effect on the expression of these receptors was observed at ML (19 h post-injection) while *dlGnRHR-II-1a* and *dlGnRHR-II-2b* did not exhibit melatonin effects at any of the sampling times (Figure 2).

## 3. Discussion

In this work, we have determined the mRNA expression of *GnRH-1*, *GnRH-2* and *GnRH-3* and five *GnRH* receptors in the brain of the European sea bass by using real-time quantitative PCR. Our findings revealed the existence of day–night variations in the brain expression of *GnRH-1* and *GnRH-3* as well as of *dlGnRHR-II-1c* and *dlGnRHR-II-2a* receptors, which exhibit higher mRNA levels at mid-light in relation to dusk and mid-dark phases of the photocycle Although tempting, extrapolation of changes in transcript levels into similar changes in the biologically active peptide/protein levels requires further analysis. It is worth mentioning that GnRH-1 and GnRH-3 represent the two hypophysiotrophic forms of sea bass, while GnRH-2 does not reach the pituitary [24]. GnRH-1 and GnRH-3 fibers innervate profusely gonadotrophic and somatotrophic cells, but were also evident close to the prolactin, somatolactin and MSH cells [43]. A previous study performed in sea bass also reported daily rhythms in the GnRH-1 protein levels, but in this case in the pituitary [37]. Both pituitary contents [37] and brain expression levels [present work] of the GnRH-1 exhibited lower values during the night in sea bass. Daily changes in mRNA levels of different *GnRH* forms have been observed in another perciform species, the gilthead sea bream *Sparus aurata*[44]. These findings were consistent with our results, since the expression levels were also higher at daytime, but in the case of the sea bream, *GnRH-2* form also exhibited daily variation. This discrepancy could reflect real species-specific differences but also differences related to the season or reproductive stage of the animals used (winter/spawning animals for sea bream *vs.* spring/resting animals for sea bass). It is also possible that daily variations of *GnRH-2* expression are shifted in relation to the other *GnRH* forms and appear masked by using this experimental design.

To the best of our knowledge, our results represent the first report of a day–night fluctuation in the brain expression levels of fish *GnRH* receptors. These day–night variations were only found in two of the five receptors analyzed suggesting that they appear subjected to different regulatory mechanisms. Previously, *GnRH* receptors expression was revealed to follow daily fluctuation throughout the rat estrus cycle, peaking generally at daytime [45]. Moreover, it has been described that pulsatile GnRH can up-regulates the expression of its own receptor mRNA in rat [46]. It remains to be determined if this *GnRH* receptor mRNA fluctuation is regulated hormonally by its ligand in sea bass and if day–night variations in receptor transcript levels are also correlated with daily differences in the amount of functional receptor proteins. In sea bass, all GnRH-1 fibers are directed to the pituitary while GnRH-2 and GnRH-3 innervate profusely the brain and represent the potential ligands for brain GnRH receptors [24]. Interestingly, dlGnRHR-II-2b is the receptor subtype that shows the highest affinity for GnRH-2 [26,47] and none of these genes exhibit day–night variation in transcript levels.

**Figure 3:**
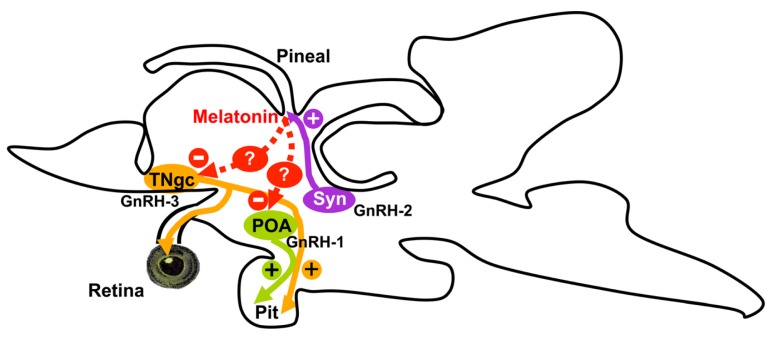
The proposed mechanism of interactions of different GnRH systems with photoreceptive organs (pineal and retina) and pituitary in the European sea bass. GnRH 1 and GnRH 3 represent the hypophysiotrophic forms (+) whereas GnRH-2 does not send projections to the pituitary [24]. Moreover, GnRH-2 and GnRH-3 fibers reach the pineal organ and the retina, respectively [26,52]. In the pineal organ, GnRH-2 stimulates (+) melatonin secretion [26] that, in turn, reduces (−) *GnRH-1* and *GnRH-3* transcript levels [present study]. These inhibitory actions of melatonin are probably mediated indirectly by interneurons (represented by “?” in figure) because the distribution of melatonin receptor-expressing cells [36] does not match with that of GnRH-1 and GnRH-3 cells.

We have also tested the effects of melatonin injection in the brain expression of *GnRHs* and *GnRH* receptors in sea bass. The melatonin dose used in the present study was in the range of those chosen in previous studies carried out in fish [9,48,49]. Our experimental design consisted in a single intraperitoneal injection of melatonin during the late-light phase of the day–night cycle, to coincide with the natural nocturnal rise of melatonin [7,32]. In the present study, an inhibitory effect of melatonin on the nocturnal brain expression of *GnRH-1*, *GnRH-3*, and *GnRH* receptor subtypes *1c*, *2a* and *2b* was also demonstrated. The inhibitory effect of melatonin on *GnRH* gene expression has also been reported in GT1-7 GnRH-secreting neurons [6]. The central effects of melatonin on brain GnRH systems reported in the present study could be supported by the presence of melatonin receptors in neuroendocrine brain areas of the sea bass brain [36]. Nevertheless, a direct effect of the pineal hormone on GnRH cells is unlikely because GnRH and melatonin receptor-expressing cells do not appear to be co-localized in the same brain areas. These inhibitory actions of melatonin are probably mediated by interneurons (Figure 3). In sea bass, kiss1- and kiss2-immunoreactive neurons were identified in the lateral tuberal nucleus and the parvocellular preoptic nucleus, respectively [50], two cell masses that also express melatonin receptors [36]. If kisspeptin cells are mediating these inhibitory actions of melatonin on GnRH systems remains to be deciphered.

With the exception of *dlGnRHR-II-2b*, the inhibitory effect of melatonin was evident on *GnRH* forms and *GnRH* receptors that exhibited significant day–night fluctuations in their expression, suggesting that exogenous melatonin could be reinforcing physiological mechanisms already established. In this way, exogenous melatonin appears to determine the maintenance of the early night conditions, probably because plasma melatonin levels remain elevated during a longer period of time. As a result, the expression levels of genes that exhibit day–night variations remain low in melatonin-injected animals at MD (7 h after the injection), as in the control group at dusk. It appears that melatonin injection is mimicking and enhancing physiological inhibitory actions of endogenous melatonin on particular *GnRH* and *GnRH* receptor genes. In any case, these effects seem to be transitory because no difference in the expression of any of the genes analyzed was found at ML (19 h post-injection) between controls and melatonin-injected animals. Only in the case of the receptor dlGnRHR-II-2b, the exogenous melatonin administration inhibits its brain expression levels at mid-dark phase of the photocycle, even thought this receptor subtype does not show apparent physiological day–night variations. As it was indicated above, GnRH-2 is the ligand that shows the highest affinity for dlGnRHR-II-2b receptor [26,47] and, like its preferential receptor, it does not exhibit significant variations along the daytimes analyzed. It is noteworthy that this receptor is strongly expressed in the sea bass pineal organ [26], which is the main physiological source of melatonin. We have also demonstrated that GnRH-2 reaches the pineal organ of sea bass and can stimulate *in vivo* and *in vitro* melatonin secretion [26]. Therefore, this inhibitory effect of melatonin on *dlGnRHR-II-2b* expression could be a part of a regulatory feedback mechanism.

The results obtained in our study in sea bass suggest that melatonin, through its transcriptional effects on hypophysiotrophic GnRH systems, could act as a synchronizer of the hormonal rhythms at precise time of the day and, presumably, of the year. In this sense, not only daily rhythms, but also seasonal rhythms of melatonin and melatonin receptors have been reported in sea bass [32,35]. It is interesting to note that natural reproduction occurs in sea bass under short photoperiod regimes of winter, when circulating plasma melatonin levels are low, but it ceases in spring when day-length and plasma melatonin levels increase considerably [32]. This timing would be, therefore, critical for the control of the reproductive cycle in this species. Consequently, photoperiod manipulation in sea bass affected both plasma melatonin and receptor mRNA oscillations, and significantly altered the circadian and annual profile of several reproductive hormones, preventing the achievement of the reproductive process [35,37,42,51].

We propose that melatonin could modulate the reproductive axis of sea bass at the central level through its transcriptional actions on particular brain GnRH systems (*i.e.*, GnRH-1 and GnRH-3 but not GnRH-2) and receptors (Figure 3). It should be noted that GnRH-2 does not reach the sea bass pituitary but innervates profusely the brain and the pineal organ of sea bass, where it is able to stimulate melatonin secretion [24,26] (Figure 3). Taking into account that GnRH-1 and GnRH-3 represent the hypophysiotrophic GnRH forms in sea bass our results suggest that these melatonin effects at the brain level could also have a functional correlate in the pituitary gland and in gonadotrophin secretion. However, an indirect action of melatonin is also expected to operate at central level, likely via interneurons, even thought which neuronal population plays this role remains to be elucidated (Figure 3).

## 4. Material and Methods

### 4.1. Animals

Male specimens of European sea bass, *Dicentrarchus labrax* (<200 g in body mass) were used in the present study. Animals were kept in running seawater at a temperature and salinity of 19 ± 1 °C and 39 ppt, respectively, in indoor facilities from the Laboratorio de Cultivos Marinos (CASEM, University of Cadiz, Puerto Real, Spain) receiving natural environmental light. Animals were fed at libitum once per day. All animals were treated in agreement with the European Union regulation (EC Directive 86/609/EEC) concerning the protection of experimental animals. Animal experimental protocols were approved by the Animal Care and Use Committee of the University of Cadiz.

### 4.2. Experimental Procedure

This experiment was carried out in spring (Mid-March, sunrise, 07:26; sunset, 19:39, GMT + 1). Fourteen sea bass males were intraperitoneally injected with melatonin (Sigma Aldrich Chemicals, St. Louis, MO, USA), one hour before dusk. The dose injected was 0.5 μg/g of body mass of melatonin dissolved in vehicle solution (saline solution containing 1% ethanol). A control group (*n* = 21) of fish belonging to the same stock, was injected with the vehicle solution. At dusk (one hour after the injection), 7 fish of the control group were sacrificed and their brain were quickly extracted, frozen in liquid nitrogen and stored at −80 °C until used for RNA extraction. The same procedure for brains collection was repeated at mid-dark (MD, seven hours after the injection) and at mid-light phase of the photocycle (ML, nineteen hours after the injection) of the following day, with 7 fish sacrificed per group and experimental condition (control *vs.* melatonin) at each sampling point.

### 4.3. Quantitative Real Time PCR Assays in Sea Bass Brain

Total RNA was extracted from each brain using EUROzol (EuroClone, Siziano, Italy) according to the manufacturer’s instructions. Total RNA (1 μg) was retro-transcribed and genomic DNA removed (QuantiTect^®^ Reverse Transcription Kit, Qiagen, Hilden, Germany). Real-time gene expression analysis was performed in a Chromo 4™ Four-Color Real-Time System (Biorad, Alcobendas, Spain), using 18*S* for normalization. 18*S* expression did not exhibit statistical differences between samples and treatment groups, revealing that it represents an adequate housekeeping gene for this study. PCR reactions were developed by duplicated in a 25 μL volume using cDNA generated from 1 μg of RNA, iTaq™ SYBR^®^ Green Supermix with ROX (Biorad, Alcobendas, Spain) and specific primers (0.4 μM, Table 1). All calibration curves exhibited slopes close to −3.32 and efficiencies around 100%. PCR reactions were performed using the following steps: 3 min at 95 °C, 30 s at 95 °C, 30 s at the annealing temperature (Table 1) and 45 s at 72 °C. The obtained PCR products were run in agarose gels and sequenced to ensure the specificity of the amplification. Besides, melting curves were analyzed for each sample, in order to confirm that only a single sequence was amplified. Negative controls included replacement of cDNA by water and the use of non retro-transcribed total RNA. The ΔΔCt method [53] was used to determine the relative mRNA expression.

### 4.4. Data Analysis

Statistical differences among control animals sampled at different daily points were determined using one-way ANOVA followed by a multiple contrast of range test (LSD). The differences in gene expression between controls and melatonin-treated animals sampled at MD (seven hours after the injection) and ML (nineteen hours after the injection) were determined using a two way ANOVA, following by a LSD test. When data did not accomplish with the requirements of the parametric ANOVA (homogeneity of variances, normality), data were analyzed using the non-parametric Kruskal-Wallis ANOVA on ranks followed by Duncan’s test. *p* < 0.05 was taken as statistical significant threshold. All statistical tests were made using the Statgraphics software.

## 5. Conclusions

We have reported the existence of day–night variations as well as melatonin inhibitory effects in the brain expression of *GnRH-1*, *GnRH-3* and several *GnRH* receptors in the European sea bass. Although correlation of changes in mRNA levels with changes in biologically active peptides/proteins remains to be revealed, these interactions between melatoninergic and GnRH systems could represent a substrate of photoperiod effects on reproductive and other rhythmic physiological events in the European sea bass.

## Figures and Tables

**Table 1:** List of primers used for the quantitative RT-PCR analysis in sea bass brain. Annealing temperatures and GenBank accession numbers are also indicated. *GnRH-R II-1a, GnRH-R II-1b*, *GnRH-R II-1c*, *GnRH-R II-2a* and *GnRH-R II-2b* correspond to different *GnRH* receptor subtypes sequences identified in sea bass. *GnRH-1*, *GnRH-2* and *GnRH-3* correspond to different GnRH forms present in this species. The reference gene used as housekeeping was *18S*.

Gene	Forward primer sequence	Reverse primer sequence	Annealing temperature	GenBank accession no.
*GnRH-1*	qPCR-GnRH-1-F: GGTCCTATGGACTGAGTCCAGG	qPCR-GnRH-1-R: TGATTCCTCTGCACAACCTAA	60 °C	AF224279
*GnRH-2*	qPCR-GnRH-2-F: GTGTGAGGCAGGAGAATGCA	qPCR-GnRH-2-R: CTGGCTAAGGCATCCAGAATG	60 °C	AF224281
*GnRH-3*	qPCR-GnRH-3-F: TGTGGGAGAGCTAGAGGCAAC	qPCR-GnRH-3-R: GTTTGGGCACTCGCCTCTT	60 °C	AF224280
*GnRH-R II-1a*	qPCR-1a-F: CTCTGGCTATCAATAAGGC	qPCR-1a-R: CTCGGGATGGATGATGGT	59 °C	AJ419594
*GnRH-R II-1b*	qPCR-1b-F: CTGCTGATGTTCTTGAAACTGG	qPCR-1b-R: GAAGTTCTCTGGCACTGTGATG	64 °C	AJ606686
*GnRH-R II-1c*	qPCR-1c-F: TGATGGTGGCGTGGACTA	qPCR-1c-R: GAGTAAAGTTTGCTGGATAAG	59 °C	AJ606684
*GnRH-R II-2a*	qPCR-2a-F: TGACGCTGTATGTCTTCCC	qPCR-2a-R: CATCCGGGCTTTGGGTAT	59 °C	AJ606683
*GnRH-R II-2b*	qPCR-2b-F: AGACTCTGAAGATGACGGTGGT	qPCR-2b-R: AGTGAAGCGTCTCTTCTCATCC	64 °C	AJ606685
*18S*	qPCR-18S-F: GCATGGCCGTTCTTAGTTGGT	qPCR-18S-R: GCATGCCGGAGTCTCGTT	48 °C	AY831388
